# Circular RNAs: Biomarkers of cancer

**DOI:** 10.1002/cai2.28

**Published:** 2022-09-21

**Authors:** Jingyi Cui, Meng Chen, Lanxin Zhang, Sida Huang, Fei Xiao, Lihui Zou

**Affiliations:** ^1^ The Key Laboratory of Geriatrics, Beijing Institute of Geriatrics, Institute of Geriatric Medicine, Chinese Academy of Medical Sciences Beijing Hospital/National Center of Gerontology of National Health Commission Beijing China; ^2^ Clinical Biobank, Beijing Hospital, National Center of Gerontology, National Health Commission, Institute of Geriatric Medicine Chinese Academy of Medical Sciences Beijing China; ^3^ Key Laboratory for National Cancer Big Data Analysis and Implement, National Cancer Data Center, National Cancer Center/National Clinical Research Center for Cancer/Cancer Hospital Chinese Academy of Medical Sciences and Peking Union Medical College Beijing China; ^4^ Department of Public Policy Cornell University Ithaca New York USA

**Keywords:** circRNA, cancer, biomarker

## Abstract

Circular RNAs (circRNAs) are a class of single‐stranded closed RNAs that are produced by the back splicing of precursor mRNAs. The formation of circRNAs mainly involves intron‐pairing‐driven circularization, RNA‐binding protein (RBP)‐driven circularization, and lariat‐driven circularization. The vast majority of circRNAs are found in the cytoplasm, and some intron‐containing circRNAs are localized in the nucleus. CircRNAs have been found to function as microRNA (miRNA) sponges, interact with RBPs and translate proteins, and play an important regulatory role in the development and progression of cancer. CircRNAs exhibit tissue‐ and developmental stage–specific expression and are stable, with longer half‐lives than linear RNAs. CircRNAs have great potential as biomarkers for cancer diagnosis and prognosis, which is highlighted by their detectability in tissues, especially in fluid biopsy samples such as plasma, saliva, and urine. Here, we review the current studies on the properties and functions of circRNAs and their clinical application value.

## Graphical abstract

This study demonstrates that circular RNAs (circRNAs) have great potential as biomarkers for cancer diagnosis and prognosis, which is highlighted by their detectability in tissues and fluid biopsy samples. CircRNAs may act as biomarkers in cancers including glioma, bladder cancer, breast cancer, gastric cancer, hepatocellular carcinoma, colorectal cancer, lung cancer, kidney cancer, pancreatic cancer, and leukemia.

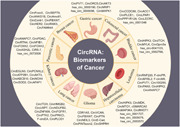

**Abbreviations**
BC: breast cancer
CCA: cholangiocarcinoma
ccRCC: clear cell renal cell carcinoma
CircRNA: circular RNA
CiRNA: intronic circRNA
CRC: colorectal cancer
EcRNA: exonic circRNA
EIcRNA: exon‐intron circRNA
GC: gastric cancer
HCC: hepatocellular carcinoma
HPSCC: hypopharyngeal squamous cell carcinoma
ICC: intrahepatic cholangiocarcinoma
IRES: internal ribosome entry site
LSCC: laryngeal squamous cell carcinoma
MB: medulloblastoma
miRNA: microRNA
m^6^A: N6‐methyladenosine
NSCLC: nonsmall cell lung cancer
ORF: open reading frame
OSCC: oral squamous cell carcinoma
PC: prostate cancer
RBP: RNA‐binding protein
TNBC: triple negative breast cancer

## 1 BACKGROUND

Circular RNAs (circRNA) exist widely in eukaryotic cells [1]. CircRNAs are produced by back‐splicing of the upstream 5′ splice donor site with the upstream 3′ splice acceptor site and lack 5′ N7‐methylguanosine caps and 3′ polyadenylated tails. CircRNAs are not easily degraded by RNA exonuclease (such as RNase R) and are more stable than linear RNA. The circRNA formation models include intron‐pairing‐driven circularization, RBP‐driven circularization, and lariat‐driven circularization [2, 3]. CircRNAs function as microRNA (miRNA) sponges, protein scaffolds, or by encoding proteins. Numerous studies have demonstrated that circRNAs play a crucial role in cancer and can serve as potential cancer biomarkers and therapeutic targets [4, 5].

In this study, we discuss the biogenesis, properties, and functional mechanisms of circRNAs and the clinical potential of these circRNAs as biomarkers for cancer diagnosis and prognosis. We summarize current research on the functions of circRNAs in different tissues and body fluids as biomarkers for cancer. We also review the major challenges in the clinical application of circRNAs as effective therapeutic targets for cancer treatment.

## 2 PROPERTIES OF CircRNAs IN EUKARYOTIC CELLS

CircRNAs are produced from precursor mRNAs by back‐splicing through a mechanism that competes with canonical splicing [6]. CircRNAs have a stable structure and exhibit tissue‐ or developmental stage‐specific expression. These molecules also have a high degree of conservation among different species. Furthermore, circRNAs are closely related to the occurrence and development of diseases, which makes them a suitable candidate for serving as biomarkers for cancer [7].

### 2.1 Stable structure

CircRNAs are stable in cells because of their covalently closed circular structure and the lack of 5′ N7‐methylguanosine caps and 3′ polyadenylated tails, making them resistant to digestion by ribonucleases. While mRNAs typically have a half‐life of 4.0–7.4 h, most circRNAs have a half‐life of 18.8–23.7 h. Some circRNAs have a half‐life of 10‐times that of linear RNAs [8]. CircRNA can persist stably in blood, urine, saliva, and other body fluids and thus are suitable candidates for biomarkers.

### 2.2 Tissue‐specific and developmental stage‐specific expression

CircRNAs are found in most human tissues and are notably abundant in the brain [9]. With the rapid development of next‐generation sequencing, many circRNAs have been identified, most of which are only found in specific tissues. Studies have shown that circRNAs exhibit tissue expression specificity and developmental stage specificity. The expression of several circRNAs varies with developmental stage. One study showed that the abundance of some circRNAs changes with age [10].

### 2.3 Evolutionary conservation across species

Recent technological advances have led to the discovery of circRNAs in human, mice, nematodes, protists plants [11] zebrafish [12], and *Drosophila* [13]. CircRNAs are highly evolutionarily conserved across species. For example, numerous circRNAs are expressed in both human and mice [10]. In addition, the expression of circRNAs in mammals is relatively conserved, especially in the brain tissue of mammals.

## 3 FUNCTIONAL MECHANISMS OF CircRNAs

**Figure 1:**
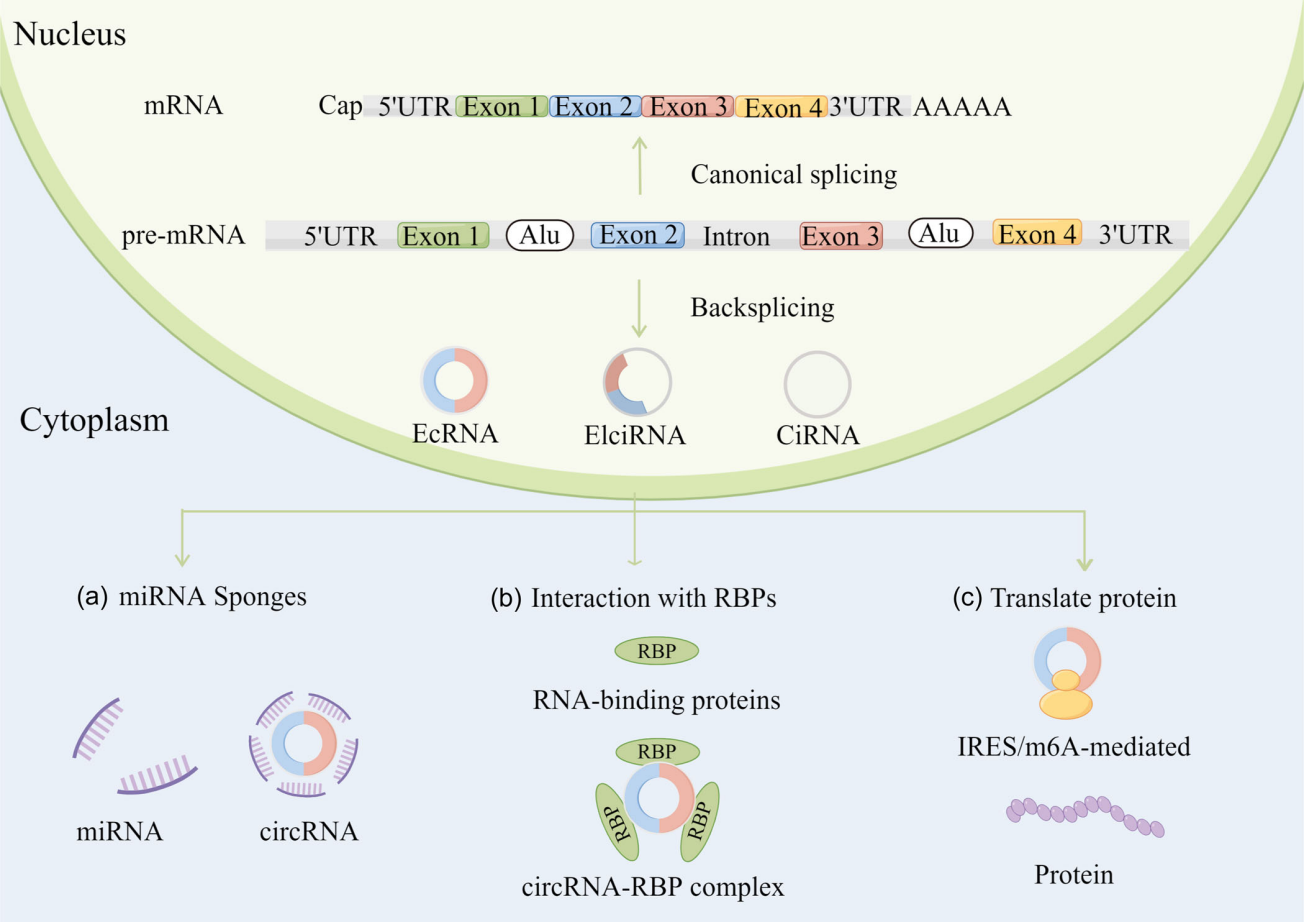
Biogenesis and functional mechanisms of circular RNA (circRNAs). CircRNAs are produced from premessenger RNA by a noncanonical process called back‐splicing. Backsplicing and canonical splicing compete with each other to produce circRNAs or mRNAs, respectively. CircRNAs are classified into three types based on their sequence origins: exonic circRNAs (EcRNA), intronic circRNAs (CiRNA), and exon‐intron circRNAs (EIcRNA). (a) CircRNAs can function as miRNA sponges to regulate gene expression. (b) CircRNAs can interact with RBPs and act as protein decoys or scaffolds to regulate their functions. (c) CircRNAs are translated through IRES‐mediated or m^6^A‐mediated translation. This figure was generated by Figdraw (https://www.figdraw.com/static/index.html).

In recent years, a large number of studies have shown that circRNAs are closely related to biological growth and development, immune response, disease occurrence and development. The biological functional mechanisms of circRNA mainly include three types: acting as miRNA sponges (Figure 1a), interacting with RBPs (Figure 1b), and translating proteins (Figure 1c).

### 3.1 miRNA sponges

CircRNAs play important roles in posttranscriptional gene regulation through acting as miRNA sponges. CircRNAs prevent miRNAs from regulating their target genes, thereby acting as competing endogenous RNAs. CDR1AS, or ciRS‐7, contains more than 70 miR‐7 target sites and functions as a miRNA sponge in specific tissues [14]. CircHIPK3 also sponges multiple miRNAs; it binds to tumor‐suppressive miR‐124, and overexpression of this circRNA promotes cell–cell growth [15, 16].

### 3.2 Interaction with RBPs

Some circRNAs bind to RBPs and act as protein decoys or scaffolds for protein assembly. Wang et al. identified a cancer‐related circRNA, circMTCL1, that functions in the occurrence and progression of advanced laryngeal squamous cell carcinoma (LSCC) through its protein sponging properties. It promotes ubiquitin degradation by interacting with complement C1q‐binding protein and activates Wnt/β‐catenin signaling pathway [17]. Yang et al. demonstrated that circWSB1, a novel hypoxia‐responsive circRNA, competitively binds to ubiquitin‐specific protease 10 in breast cancer (BC) cells. This binding prevented the interaction between p53 and ubiquitin‐specific protease 10 in BC cells, resulting in p53 degradation and BC development [18].

### 3.3 Translation of circRNAs

Some polypeptides and proteins translated by circRNAs have pathogenic or inhibitory effects in various diseases. Du et al. discovered an upregulated circRNA (circNlgn) in congenital heart disease patients. CircNlgn encodes a novel peptide Nlgn173 with a 9‐amino‐acid nuclear localization motif. Nuclear localization of Nlgn173 is promoted by its binding to LaminB1 [19]. There are three main mechanisms in the translation of circRNAs: internal ribosome entry site (IRES)‐mediated circRNA translation, N6‐methyladenosine (m^6^A) modification‐mediated circRNA translation, and rolling circle translation.

#### 3.3.1 IRES‐mediated circRNA translation

Some circRNAs are translated in an IRES‐mediated manner, through a mechanism distinct from cap‐dependent translation. The IRES sequence has a special structure that binds to the ribosome and directly initiates translation. CircRNAs do not have a 5′ cap structure, and thus the IRES sequence is essential for their protein‐encoding ability. For example, circ‐ZNF609 contains an IRES that allows for cap‐independent translation, indicating circRNAs containing IRESs can be translated into protein [20].

#### 3.3.2 M^6^A modification‐mediated circRNA translation

m^6^A modification is one of the most common RNA modifications. Yang et al. conducted methylated RNA immunoprecipitation sequencing and found that approximately 13% of all circRNAs have m^6^A modifications [21]. Another mechanism of circRNA translation involves m^6^A modifications, which mediate circRNA translation in eukaryotic cells. M^6^A‐initiated translation is dependent on eukaryotic translation initiation factor 4, gamma 2 or eukaryotic initiation factor 3 and the YTH domain family protein 3. Some m^6^A‐modified short RNA elements that are enriched in circRNA sequences, which can initiate cap‐independent translation [22]. Li et al. discovered a novel oncogenic circRNA named circARHGAP35, which has a 3867 nt open reading frame (ORF) with an m^6^A‐modified start codon. circARHGAP35 produces a truncated protein with four FF domains that accelerates the growth of cancer cells by interacting with TFII‐I in the nucleus [23].

#### 3.3.3 Rolling circle translation of circRNA

Rolling circle amplification is an isothermal and enzymatic process mediated by specific DNA polymerases, which is considered to be a mechanism of circRNA translation. Without a stop codon, a start codon and an infinite ORF are sufficient for rolling circle translation of circRNAs. Liu et al. used an RNA cyclase ribozyme mechanism to generate a circularized mRNA. The resulting circRNA acted as a translation template for the production of long peptides with tandem repeats and encoded the spider silk proteins FSLP and MaSp1 [24].

## 4 CircRNAs AS BIOMARKERS OF CANCER

**Figure 2:**
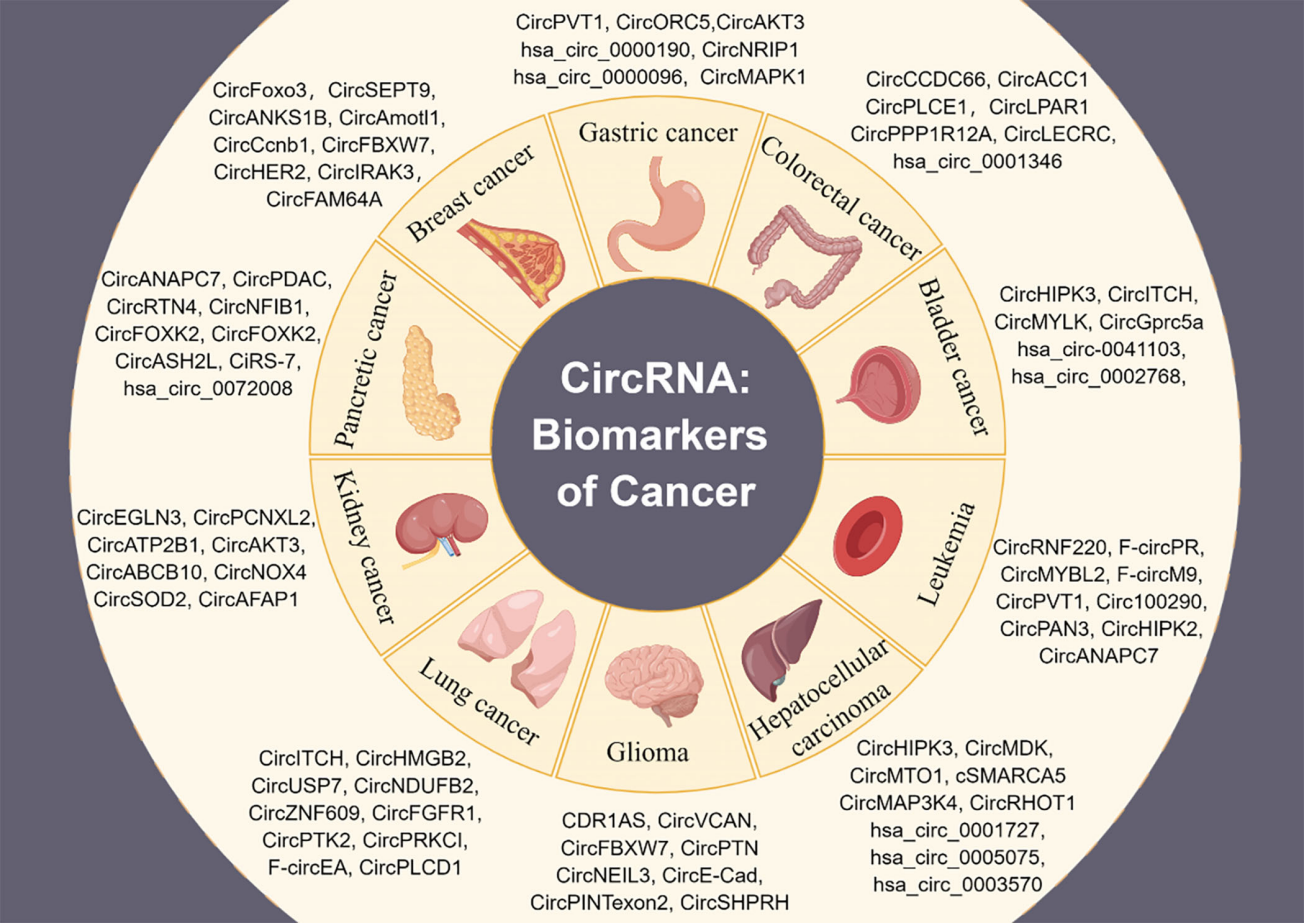
Dysregulated circular RNA (circRNAs) in common cancers. circRNAs may act as biomarkers in cancers including glioma, bladder cancer, breast cancer, gastric cancer, hepatocellular carcinoma, colorectal cancer, lung cancer, kidney cancer, pancreatic cancer, and leukemia. This figure was generated by Figdraw.

The high abundance and stability of circRNAs combined with their unique expression signatures in cancer highlight their potential as diagnostic and prognostic biomarkers for cancer [22] (Figure 2). Multiple circRNAs have been found to persist stably and at high levels in human tissues and fluids such as plasma, urine, saliva, bile, and gastric juice (Table 1).

**Table 1:** A list of circular RNA (circRNAs) and their functions in different cancers.

Cancer	CircRNA	Sample	Function	Ref.
MB	circRMST	Tissue	Classification	[25]
GC	hsa_circRNA_102958		Diagnosis	[26]
	circORC5		Inhibits cancer progression	[27]
ICC	CircACTN4		Promotes proliferation and metastasis	[28]
CRC	circPLCE1		Inhibits cancer progression	[29]
BC	circSEPT9		Promotes the carcinogenesis and development	[30]
Glioma	circNEIL3		Promotes cancer progression and macrophage immunosuppressive polarization	[31]
HPSCC	circCUX1		Confers radioresistance	[32]
HCC	circMDK		Promotes cancer progression	[33]
	circMAP3K4		Inhibits apoptosis	[34]
NSCLC	circHMGB2		Remodels the tumor microenvironment and regulates anti‐PD1 resistance	[35]
	circIGF2BP3		Promotes tumor immune evasion	[36]
	circUSP7		Induces resistance to anti‐PD1 immunotherapy	[37]
CRC	CircLPAR1	Plasma	Inhibits tumorigenesis	[38]
GC	hsa_circ_0000190		Diagnosis	[39]
PC	circPDLIM5, circSCAF8, circPLXDC2, circSCAMP1, circCCNT2	Urine	Diagnosis	[40]
ccRCC	circEGLN3, circSOD2		Diagnosis	[41]
OSCC	hsa_circ_0001874, hsa_circ_0001971	Saliva	Diagnosis	[42]
CCA	circCCAC1	Bile	Promotes CCA tumorigenesis and metastasis	[43]
GC	hsa_circ_0014717	Gastric juice	Diagnosis	[44]

### 4.1 CircRNAs as diagnostic biomarkers

Because of the specific expression and long‐term stability of circRNAs, some circRNAs may be potential biomarkers for tumor diagnosis. Several studies showed that circRNA expression profiles distinguished various pediatric central nervous system cancers such as medulloblastoma subgroups [45]. For example, circRMST distinguishes WNT‐type medulloblastoma from other types of medulloblastoma, suggesting that circRMST may be an effective marker for medulloblastoma classification [25]. Moreover, the expression level of hsa_circRNA_102958 in GC tissues was positively associated with TNM stage (*p* = 0.032). The area under the ROC curve was 0.74. The results indicated that hsa_circRNA_102958 has potential as a diagnostic biomarker for gastric cancer [26].

### 4.2 CircRNAs as prognostic biomarkers and therapeutic targets

CircRNAs exhibit distinctive expression patterns in tumor tissues, indicating their potential as biomarkers in tissue biopsy for cancer prognosis. Fan et al. found that Mettl14 upregulates the m^6^A modification level of circORC5 and inhibits the expression of circORC5, which regulates the miR‐30c‐2/AKT1S1 axis to inhibit gastric cancer progression [27]. High expression of circACTN4 in intrahepatic cholangiocarcinoma (ICC) promotes tumor proliferation and metastasis by competitively binding to miR‐424‐5p to upregulate Yes‐associated protein 1 expression. circACTN4 also recruits Y‐box binding protein 1, a positive regulator of the Wnt/β‐catenin signaling pathway, to transcriptionally activate Frizzled‐7 [46]. Another study showed that circACTN4 coordinates the activation of Hippo and Wnt/β‐catenin pathways, which suggests that circACTN4 has the potential to act as a prognostic marker and therapeutic target for ICC [28]. Liang et al. found that circPLCE1 downregulation is related to advanced clinical stage of colorectal cancer (CRC) and poor survival of patients. The novel NF‐κB regulator circPLCE1‐411 encoded by circPLCE1 reduced NF‐κB nuclear translocation in CRC cells, resulting in the inhibition of tumor proliferation and metastasis [29]. CircSEPT9 is upregulated in triple‐negative breast cancer (TNBC), and Kaplan–Meier survival analysis showed a positive correlation between circSEPT9 and advanced clinical stage and poor prognosis, suggesting that circSEPT9 may be a prognostic biomarker and therapeutic target for TNBC [30]. CircNEIL3 is upregulated in glioma and positively correlates with malignant progression, which suggests its potential as a cancer biomarker [31]. In hypopharyngeal squamous cell carcinoma (HPSCC) patients resistant to radiotherapy, the expression of circCUX1 was upregulated and positively correlated with poor survival. CircCUX1 knockdown increased the radiotherapy sensitivity of HPSCC cells, indicating circCUX1 as a possible therapeutic target for radiation tolerance in HPSCC patients [32]. CircMDK is novel oncogenic circRNA in hepatocellular carcinoma (HCC) with significantly increased expression in tumors. CircMDK promotes HCC progression via the miR‐346/miR‐874‐3p‐ATG16L1 axis and may be a therapeutic target for HCC [33]. M^6^A‐modified circMAP3K4 encodes a novel peptide, circMAP3K4‐455 aa, which prevents apoptosis in HCC [34]. Targeting circMAP3K4‐455 aa may be a new therapeutic strategy for HCC.

Furthermore, thousands of circRNAs participate in the regulation of innate immune responses [47]. The expression of circHMGB2 is markedly upregulated in nonsmall cell lung cancer (NSCLC) and promotes the progression of NSCLC by remodeling the tumor microenvironment and regulating anti‐PD1 resistance. Survival analysis indicated that circHMGB2 is an independent indicator of poor prognosis in NSCLC patients, providing a new strategy for NSCLC treatment [35]. Interestingly, current studies have shown that circRNAs could function as competing endogenous RNAs to regulate the PD‐L1 expression in many tumors [48]. Liu et al. discovered that circIGF2BP3 promotes the deubiquitination of PD‐L1, resulting in immune escape from CD8^+^ T cell‐mediated attack in NSCLC [36]. Similarly, exosomal circUSP7 derived from NSCLC cells induces resistance to anti‐PD1 immunotherapy by promoting CD8^+^ T cell dysfunction in NSCLC, providing a rationale for improving anti‐PD1 treatment efficacy in NSCLC [37].

## 5 METHODS FOR CIRCRNA DETECTION

Many methods have been established to examine the expression and biological functions of circRNA. High‐throughput RNA‐seq and microarrays are applied to acquire circRNA profiles [49]. In recent years, researchers have successively proposed new strategies to identify the full‐length and specific alternative splicing events of circRNAs with Nanopore sequencing. Compared with next‐generation sequencing, the optimized nanopore sequencing system and bioinformatics methods can greatly improve the detection efficiency and accurately quantify the expression of circRNAs. Moreover, nanopore sequencing can achieve the full sequence assembly of circRNAs with a length of 100 bp–5 kb [50, 51, 52, 53]. Real‐time quantitative polymerase chain reaction and Northern blot are the primary methods used to analyze circRNA expression. The localization of circRNAs can be detected by fluorescence in situ hybridization. The interactions of circRNA‐miRNA and circRNA‐protein are identified using circRNA database predictions, RNA immunoprecipitation, double luciferase reporter gene assays, and RNA pull‐down combined with mass spectrometry. Additionally, bioinformatics analyses are used to predict ORFs, m^6^A modifications, and IRES in circRNAs to examine the capacity of circRNA to translate protein.

### 5.1 CircRNAs in liquid biopsy

CircRNAs are enriched and stabilized in body fluids; they may serve as more convenient and noninvasive liquid biopsy biomarkers to reflect the status of diseases at early and late stages than traditional biopsy markers in tumor tissue. Conventional biomarkers derived from body fluids are commonly used in cancer diagnosis and prognosis; however, their low sensitivity and specificity restrict their widespread application in cancer screening. Many circRNAs exist stably not only in tumor tissues and cells but also in body fluids such as blood, urine, saliva, and bile. The expression patterns of circRNAs showed significant differences between cancer patients and healthy controls, suggesting that circRNAs in body fluids may serve as novel biomarkers for cancer diagnosis and prognosis.

### 5.2 CircRNAs in blood

Blood is the most common body fluid used for liquid biopsy. CircRNAs are aberrantly expressed in human cancer and have been detected in different blood components, including blood cells, plasma, and serum. Zheng et al. identified a CRC‐specific exosomal circRNA (circLPAR1) among healthy controls, precancer individuals, and CRC patients. CircLPAR1 expression in plasma exosomes decreased markedly as CRC progressed but quickly recovered after surgery. Combined with traditional tumor biomarkers CEA and CA19‐9, exosomal circLPAR1 shows specificity and improved diagnostic performance in the diagnosis of CRC (AUC 0.875). These studies showed that plasma exosomal circLPAR1 act as a diagnostic marker of CRC [38]. Roy et al. demonstrated a panel of 8 circRNAs that may successfully distinguish gastric cancer (GC) patients from healthy controls and may be utilized as diagnostic biomarkers for the early identification of GC [54]. hsa_circ_0000190 is significantly downregulated in GC patients compared with healthy controls, suggesting it may be a promising biomarker for GC [39].

### 5.3 CircRNAs in urine

Urine detection is noninvasive, does not need to be repeated, and easily integrates into the clinical workflow. Some circRNAs are packaged into small extracellular vesicles, which are released into the extracellular space. CircRNA concentrations in exosomes are higher than those in exosome‐secreting cells. Kidney tissue has been reported to release exosomes, which raises the possibility that circRNAs packaged into exosomes in urine may provide specific information about the constitution of the entire nephron. Therefore, circRNAs in urine could be a promising biomarker for kidney disease. Hutchins et al. discovered 965 characteristic circRNAs by using RNA sequencing from 114 urine and 134 plasma samples of 54 healthy male athletes [55]. A urine extracellular vesicle circRNA classifier including circSCAMP1, circPLXDC2, circSCAF8, circPDLIM5, and circCCNT2 could distinguish high‐grade prostate cancer (PC) from low‐grade PC or benign prostatic hyperplasia [40]. Moreover, the expression levels of circEGLN3 and circSOD2 are lower in the urine of clear cell renal cell carcinomas (ccRCC) patients [41]. These studies demonstrated the levels of circRNAs in urine have great potential for tumor detection.

### 5.4 CircRNAs in other body fluids

Although saliva, bile, or gastric juice/wash are affected by many physiological factors, they have been regarded as ideal biofluids for disease detection because analyses of these fluids are easy, quick, cheap, and noninvasive. Bahn et al. discovered more than 400 circRNAs in saliva using a customized bioinformatics method [56]. Hsa_circ_0001971 and hsa_circ_0001874 were correlated with TNM staging of oral squamous cell carcinoma (OSCC). The ROC curve of hsa_circ_0001971 combined with hsa_circ_0001874 reached 0.922. This combination showed good diagnostic specificity and sensitivity in distinguishing OSCC patients from oral leukoplakia patients. Hsa_circ_0001874 and hsa_circ_0001971 are downregulated in OSCC patients after surgery. This study was the first to use circRNAs in saliva as diagnostic markers for OSCC patients [42]. Moreover, increased expression of circCCAC1 was discovered in bile‐resident extracellular vesicles from cholangiocarcinoma (CCA) patients. Endothelial cells receive CCA‐delivered exosomal circCCAC1, which disrupts endothelial barriers and causes angiogenesis, leading to CCA tumorigenesis and metastasis [43]. The high specificity of gastric juice for the stomach gives it an obvious advantage in reflecting gastric disease status, suggesting that gastric juice can be used as a noninvasive method for disease diagnosis. Shao et al. identified that hsa_circ_0014717 could persist steadily in human gastric juice, suggesting it could serve as an ideal candidate for cancer biomarkers [44].

## 6 CHALLENGES AND FUTURE PERSPECTIVES

CircRNAs are receiving increasing attention because of their tissue or cell expression specificity and complex regulatory mechanisms. They play important roles in gene expression and signaling pathways and are involved in cancer occurrence and progression. CircRNAs have shown great potential in the diagnosis and treatment of tumors. Moreover, circRNAs are more stable than linear RNAs due to their closed‐loop structures. CircRNAs are widely expressed in tissues, and some of them are expressed at higher levels than their linear transcripts. In addition, they are expressed abnormally under pathological conditions. These features of circRNAs may help to address the low specificity and sensitivity of existing conventional biomarkers.

Although many circRNAs have been identified for their potential diagnostic and prognostic value in tumors, circRNAs have not been validated for clinical applications. There are still some challenges in the process of moving from bench to bedside. First, most identified circRNAs are specifically expressed in tumor tissues, but tissue biopsy would cause some wounds to the patients. CircRNAs are stable in body fluids, suggesting they may represent a convenient and noninvasive method of detection compared with tissue biopsy. However, the current detection methods of circRNA in body fluids still have limitations and challenges. Second, the cost of detecting circRNAs in tissues or exosomes is higher than that of existing tests, limiting their regular use as biomarkers. Third, compared with studies on other RNAs, research on circRNAs is still in its infancy. The mechanisms underlying circRNA expressions, functions, and association with diseases require investigation. Fourth, the sequences of circRNAs overlap with corresponding linear transcripts, and therefore methods for detecting and quantifying circRNAs face several challenges [53]. Additionally, the development of more convenient, specific, and reliable methods to detect circRNAs is required.

With ongoing research, more circRNAs will presumably be found to play an important role in disease diagnosis, prognosis, and treatment. While studies have indicated circRNAs as promising cancer biomarkers, especially in liquid biopsy, there are still many key issues to be addressed for the application of circRNAs in clinical practice. More evidence is required to support the clinical relevance of circRNAs in specific cancers through multicenter retrospective and prospective studies in a large number of clinical samples.

## AUTHOR CONTRIBUTIONS

**Jingyi Cui**: Software; validation; writing – original draft. **Meng Chen**: Investigation; methodology. **Lanxin Zhang**: Project administration; software. **Sida Huang**: Data curation; formal analysis. **Fei Xiao**: Funding acquisition; writing – review and editing. **Lihui Zou**: Supervision; writing – review and editing.

## CONFLICTS OF INTEREST

The authors declare no conflict of interest. Professor Meng Chen, Fei Xiao are the members of the *Cancer Innovation* Editorial Board. To minimize bias, they were excluded from all editorial decision‐making related to the acceptance of this article for publication.

## ETHICS STATEMENT

Not applicable.

## INFORMED CONSENT

Not applicable.

## Data Availability

Data sharing is not applicable to this article as no new data were created or analyzed in this study.
